# Therapeutic Strategies for Relapsed or Refractory B‐Cell Acute Lymphoblastic Leukemia in Adult Patients: Optimizing the Use of Monoclonal Antibodies

**DOI:** 10.1111/ejh.14405

**Published:** 2025-03-06

**Authors:** Antonella Bruzzese, Enrica Antonia Martino, Caterina Labanca, Giulio Caridà, Francesco Mendicino, Eugenio Lucia, Virginia Olivito, Noemi Puccio, Antonino Neri, Fortunato Morabito, Ernesto Vigna, Massimo Gentile

**Affiliations:** ^1^ Hematology Unit, Department of Onco‐Hematology AO of Cosenza Cosenza Italy; ^2^ Department of Experimental and Clinical Medicine University of Catanzaro Catanzaro Italy; ^3^ Laboratory of Translational Reserach Azienda USL‐IRCSS di Reggio Emilia Reggio Emilia Italy; ^4^ Scientific Directorate Azienda USL‐IRCCS di Reggio Emilia Reggio Emilia Italy; ^5^ Gruppo Amici Dell'Ematologia Foundation‐GrADE Reggio Emilia Italy; ^6^ Department of Pharmacy, Health and Nutritional Science University of Calabria Rende Italy

**Keywords:** MoAbs, RR ALL, therapy

## Abstract

The treatment landscape for relapsed or refractory acute lymphoblastic leukemia (RR ALL) has evolved significantly with the introduction of monoclonal antibodies such as blinatumomab and inotuzumab ozogamicin. These agents have demonstrated remarkable efficacy, achieving high response rates and minimal residual disease (MRD) negativity. However, the optimal selection, sequencing, and integration of monoclonal antibodies and other modalities like standard chemotherapy or chimeric antigen receptor T‐cell therapy remain areas of active investigation. The absence of direct comparative studies has led to reliance on indirect analyses, which provide conflicting results regarding the relative benefits of inotuzumab and blinatumomab. While inotuzumab is preferred in high‐disease‐burden settings due to its cytoreductive capabilities, blinatumomab shows superior performance in low‐disease‐burden settings by leveraging preserved T‐cell function. Sequential and combination approaches, such as induction with inotuzumab followed by blinatumomab consolidation, may optimize outcomes, particularly for patients undergoing subsequent allogeneic stem cell transplantation (alloSCT). The interval between inotuzumab and alloSCT is critical to mitigate the risk of veno‐occlusive disease (VOD). Despite these advances, the prognosis for patients with high‐risk genetic lesions, such as TP53 mutations, remains poor, underscoring the need for innovative therapeutic strategies. As monoclonal antibodies increasingly move into frontline therapy, their role in relapse settings must be redefined. Future research should focus on unraveling the molecular underpinnings of resistance and refining treatment paradigms to improve survival and quality of life for patients with RR ALL.

## 1 Introduction

B cells precursor acute lymphoblastic leukemia (BCP‐ALL) is one of the most challenging diseases to manage in the onco‐hematologic field due to its biological variability and aggressive clinical behavior. Historically, various treatment strategies combining multiple cytotoxic agents have been developed to improve clinical outcomes, overcome drug resistance, and minimize overlapping toxicity [1]. These regimens aim to achieve complete disease remission—ideally with minimal residual disease (MRD) negativity—and prevent relapse while maintaining an acceptable toxicity profile. The selection of therapeutic agents, dosing schedules, and treatment duration is tailored based on individual patient factors, including age, the biological characteristics of ALL (notably Philadelphia chromosome [Ph] status), and the presence of extramedullary disease [2].

In Ph‐positive ALL, treatment strategies have rapidly evolved in recent years. Regimens incorporating Tyrosine‐kinase inhibitors (TKIs) have shown complete remission (CR) rates ranging from 90% to 95%, both when combined with different chemotherapy regimens and when administered alongside steroids alone. These regimens are associated with low toxicity and enable a greater proportion of patients to undergo allogeneic stem cell transplantation (alloSCT). Moreover, induction strategies are transitioning toward reduced‐intensity chemotherapy or even chemo‐free regimens to minimize induction‐related mortality [3, 4, 5, 6, 7, 8, 9]. Post‐remission approaches, including alloSCT and maintenance therapies, are influenced by factors such as patient age, comorbidities, and MRD levels.

For Ph‐negative ALL, young and fit patients are typically treated with pediatric‐inspired chemotherapy. However, the prognosis in adults remains inferior to that in children [10, 11], with median overall survival (OS) rates declining with age: ~93% in children, ~73% in younger adults, ~28%–50% in older adults (aged 60–69 years), and 10% to 20% in those over 70 years of age [10, 11, 12, 13].

Despite advances in therapy, 5%–10% of patients exhibit primary resistance to first‐line therapy, and 30%–60% experience relapse, most commonly within the bone marrow (BM). Extramedullary relapses, although less frequent, may become more prevalent with the increasing use of immunotherapies in earlier treatment phases [2, 13, 14, 15, 16, 17, 18].

Adult patients with relapsed or refractory (RR) ALL have a poor prognosis, influenced by both disease and patient‐specific characteristics. A large retrospective study involving 11 centers and 17 060 adult patients with RR ALL diagnosed between 1990 and 2013 reported an overall CR rate of 40% following first‐line salvage therapy, decreasing to 21% and 11% after second‐line and third‐line or later salvage therapies, respectively.

Median OS was 5.8 months for first‐line salvage, 3.9 months for second‐line salvage, and 2.9 months for third‐line or later salvage. Patients treated after 2005 showed higher CR rates and slightly improved OS compared to those treated earlier. Factors associated with better survival in first‐line salvage therapy included a longer duration of first CR, later treatment periods, younger age, and lower WBC counts (< 30 000/mL) at diagnosis [19].

For patients in second or later‐line salvage therapies, OS decreased with each subsequent line of treatment. Notably, prior alloSCT did not significantly affect OS in these cohorts [19].

In the past decade, substantial progress has been made in the treatment of RR ALL. Emerging therapeutic options, particularly monoclonal antibodies and chimeric antigen receptor T‐cell (CAR‐T) therapies, have demonstrated promising efficacy and safety profiles, offering new hope for patients with this challenging disease.

This review aims to provide a comprehensive examination of the role of monoclonal antibodies in the treatment of RR B‐cell ALL in adult patients, mainly focusing on the mechanisms of action, clinical efficacy, safety profiles, and recent advancements in the development of the most successful monoclonal antibodies, inotuzumab ozogamicin and blinatumomab. Additionally, the review will explore the challenges and future directions in integrating these monoclonal antibodies into the current treatment landscape for RR B‐cell ALL.

The literature search for this review was conducted via PUBMED, utilizing search terms specific to the treatment modalities and population of interest. Search results were limited to adult patients with B‐cell RR ALL. We selected clinical trials and retrospective analyses that had more impact on regulatory decisions or clinical practice. A summary of key clinical studies evaluating the efficacy and safety of inotuzumab ozogamicin and blinatumomab is presented in Table 1.

**TABLE 1:** Clinical trial exploring Inotuzumab or Blinatumomab in RR or MRD‐positive ALL.

Disease status	Phase study	Schedule	Patients enrolled	OS	cCR	Reference
Inotuzumab monotherapy						
RR ALL	2	Inotuzumab 1.3 to 1.8 mg/m^2^ every 3 to 4 weeks, then modified at the dosage of 0.8 mg/m^2^ on day 1 and 0.5 mg/m^2^ on days 8 and 15, every 3 to 4 weeks	90	6.2 months	58%	[20]
RR ALL	3	Investigational: Inotuzumab 0.8 mg/m^2^ on day 1; 0.5 mg/m^2^ on days 8 and 15 of each cycle (cycle 1, 21 days; subsequent cycles, 28 days; ≤ 6 cycles) Comparative: FLAG, cytarabine plus mitoxantrone, HIDAC	Investigational 141 Comparative 138	Investigational: 7.7 months Comparative: 6.7 months	Investigational: 80.7% Comparative: 29.4%	[21, 22]
MRD positive ALL	2	Inotuzumab 0.6 mg/m^2^ on day 1 0.3 mg/m^2^ on day 8 (cycle 1), and 0.3 mg/m^2^ on days 1 and 8 (cycles 2–6)	26	Not reached	69% (MRD negativity)	[23]
Blinatumomab monotherapy						
RR ALL	2	1st cohort Blinatumomab 15 μg/m^2^/day 4 over 4 weeks every 6 weeks 2nd cohort 5 μg/m^2^/day then increased to 15 μg/m^2^/day over 4 weeks every 6 weeks 3rd cohort 5 μg/m^2^/day then increased to 15 μg/m^2^/day then increased to 30 μg/m^2^/day over 4 weeks every 6 weeks	36	9.8 months	69%	[23]
RR ALL	2	Blinatumomab 9 μg/day for the first 7 days and 28 μg/day thereafter over 4 weeks every 6 weeks (up to 5 cycles) Blinatumomab 28 μg/day thereafter over 4 weeks every 6 weeks (subsequent cycles)	189	6.1 months	43%	[24]
RR Ph‐ ALL	3	Investigational: Blinatumomab 9 μg/day for the first 7 days and 28 μg/day thereafter over 4 weeks every 6 weeks for induction and consolidation, blinatumomab 4‐week continuous infusion every 12 weeks for maintenance Comparative: FLAG +/− anthracycline, HD MTX, HIDAC, clofarabine‐based regimen	Investigational: 271 Comparative: 134	Investigational: 7.7 months Comparative: 4 months	Investigational: 44% Comparative: 25%	[25]
MRD positive ALL	2	Blinatumomab 15 μg/m^2^/day over 4 weeks every 6 weeks for up to 4 cycles	116	36.5 months	78% (MRD negativity)	[26]
RR Ph + ALL	2	Blinatumomab 9 μg/day for the first 7 days and 28 μg/day thereafter over 4 weeks every 6 weeks (cycle 1) Blinatumomab 28 μg/day thereafter over 4 weeks every 6 weeks (subsequent cycles)	45	9 months	35.6%	[27]
RR ALL	1b	250 μg/500 μg group: 250 μg once daily for week 1 and 500 μg three times weekly thereafter 500 μg/1000 μg group: 500 μg for week 1 and 1000 μg three times weekly thereafter	250 μg/500 μg group: 14 500 μg/1000 μg group: 13	na	250 μg/500 μg group: 85.7% 500 μg/1000 μg group: 92.3%	[28]
Combination strategies with Inotuzumab						
RR ALL	2	Mini Hyper‐CVD plus Inotuzumab on day 3 of the first 4 courses at 1.8 to 1.3 mg/m^2^ (cycle 1) and 1.3 to 1.0 mg/m^2^ (subsequent cycles)	59	11 months	78%	[29]
RR ALL	1	DA‐EPOCH plus Inotuzumab at 3 different dosage	24	17 months	84%	[30]
RR ALL	1	Venetoclax (DL1: 100/200/200; DL2: 100/200/400) followed by Induction C1: Inotuzumab days 1, 8, 15 (0.8/0.5/0.5 mg/m^2^) and Venetoclax daily per DL days 1–21	9	na	100%	[31]
Combination strategies with Blinatumomab						
RR/MRD positive ALL	1	Venetoclax days 7 to 42 of target doses ranging between 200 and 800 mg/day and Blinatumomab 28 μg/day 4 weeks on and 2 weeks off, starting on day 1. For patients with R/R ALL, Blinatumomab 9 μg/day for the first week	7	na	RR group: 33.3% MRD positive group: 75%	[32]
Combination strategies with Inotuzumab and Blinatumomab						
RR ALL	2	Mini Hyper CVD plus Inotuzumab on day 3 of the first 4 courses at 0.6 mg/m^2^ on day 2 and 0.3 mg/m^2^ on day 8 (cycle 1) and as 0.3 mg/m^2^ on days 2 and 8 (cycles 2–4) followed by 4 cycles of blinatumomab (cycles 5–8) and maintenance with 12 POMP with one course of blinatumomab after every 3 cycles Blinatumomab was given at 9 μg/day in the first 4 days of cycle 5 then escalated to 28 μg/day	110	17 months	83%	[33]

Abbreviations: ALL: acute lymphoblastic leukemia; cCR: composite complete remission; DA‐EPOCH: etoposide, prednisone, vincristine, cyclophosphamide, doxorubicin; FLAG: fludarabine, cytarabine, granulocyte colony‐stimulating factor; HD MTX: high dose methotrexate; HIDAC: high dose cytarabine; mini HyperCVD: cyclophosphamide, dexamethasone vincristine (odd cycles), methotrexate and cytarabine (even cycles); MRD: minimal residual disease; OS: overall survival; POMP: prednisone, 6‐mercaptopurine, and methotrexate; RR: relapsed or refractory.

## 2 Monoclonal Antibodies as Single‐Agent Therapy

**FIGURE 1:**
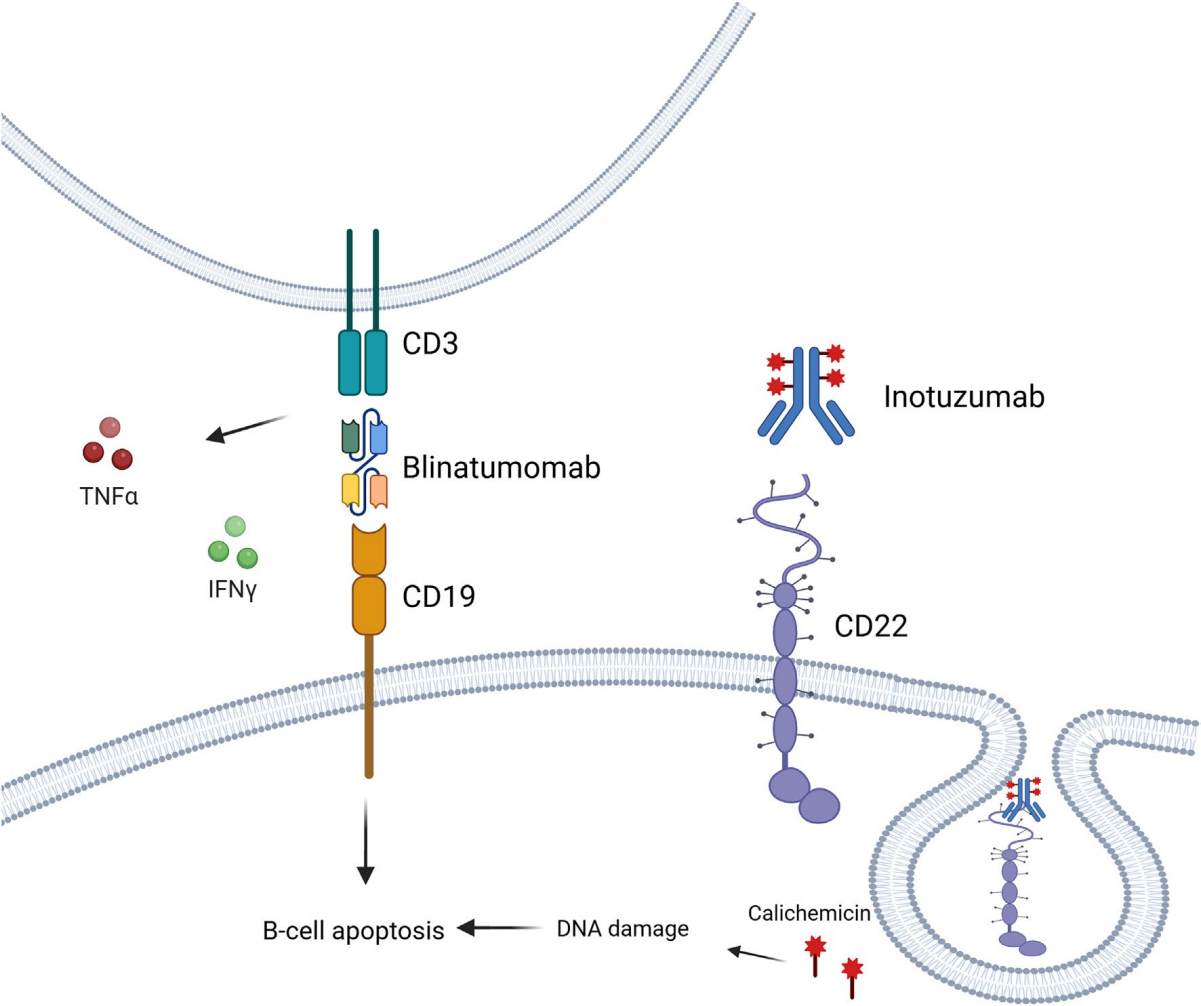
Schematic representation of the mechanisms of action of inotuzumab ozogamicin and blinatumomab in acute lymphoblastic leukemia of B‐cell origin.

Figure 1 depicts the mechanisms of action of inotuzumab ozogamicin and blinatumomab, two monoclonal antibody‐based therapies targeting distinct antigens in ALL of B‐cell origin.

### 2.1 Inotuzumab

Inotuzumab ozogamicin (CMC‐544) is a humanized monoclonal antibody (mAb) targeting CD22, conjugated to calicheamicin, a potent cytotoxic agent. Structurally, it consists of a humanized IgG4 anti‐CD22 mAb, covalently linked to calicheamicin via an acid‐labile 4‐(4′‐acetylphenoxy) butanoic acid (AcBut) linker. Mechanistically, calicheamicin binds to the minor groove of DNA, inducing double‐strand cleavage and subsequent apoptosis (Figure 1). Preclinical studies demonstrated its potent efficacy in inhibiting the growth of small but established B‐cell lymphoma (BCL) xenografts, underscoring its therapeutic potential [34, 35].

#### 2.1.1 Clinical Studies and Efficacy

A Phase 2 clinical trial assessed inotuzumab in 90 patients with RR ALL [20]. Patients were initially treated with a single intravenous dose of inotuzumab (1.3 to 1.8 mg/m^2^ every 3 to 4 weeks, *n* = 49). Subsequently, based on enhanced in vitro efficacy with prolonged exposure, a fractionated dosing regimen was employed (0.8 mg/m^2^ on day 1 and 0.5 mg/m^2^ on days 8 and 15, every 3 to 4 weeks, *n* = 41). Among these patients, 29 (32%) received inotuzumab as a first salvage treatment, 34 (38%) as a second salvage therapy, and 27 (30%) as a third or greater salvage therapy. A total of 10 patients (11%) had undergone prior alloSCT. The overall response rate (ORR) was 58%, with 19% achieving a CR, 30% CR with incomplete platelet recovery (CRp), and 9% bone marrow (BM) CR without peripheral recovery. Response rates were comparable between single dose and fractionated regimens (57% vs. 59%, respectively); however, the median OS was slightly prolonged in the fractionated dosing group (7.3 vs. 5 months). The median OS was the longest for patients treated with inotuzumab as first salvage therapy (9.2 months) compared to second (4.3 months) or third or greater salvage therapy (6.6 months). A total of 36 patients (40%) underwent alloSCT following inotuzumab treatment [20].

The fractionated dosing schedule was associated with a more favorable safety profile compared to single‐dose administration. Specifically, liver‐related adverse events (AEs) such as bilirubin and liver enzyme elevations were less frequent and less severe. Two patients treated with single‐dose inotuzumab experienced persistent liver function impairment, while liver toxicity resolved within 1–2 weeks in patients receiving fractionated dosing. Among patients undergoing alloSCT, the incidence of veno‐occlusive disease (VOD) was 17%, with a lower frequency observed in the fractionated dosing group (7%) and in patients receiving fewer alkylators in the conditioning regimen [20].

#### 2.1.2 INO‐VATE Phase 3 Study

The efficacy of inotuzumab was further validated in the INO‐VATE Phase 3 study, which randomized 326 patients with RR ALL to receive either inotuzumab ozogamicin or standard intensive chemotherapy [21].

Patients in the inotuzumab arm showed superior progression‐free survival (PFS; 5.0 vs. 1.8 months, *p* < 0.001) and OS (7.7 vs. 6.7 months, *p* = 0.04). The primary intention‐to‐treat analysis performed in the first 218 patients showed a significantly higher CR rate in the inotuzumab group (80% vs. 29.4%, *p* < 0.001), with a greater proportion of patients achieving minimal residual disease (MRD) negativity (78.4% and 28.1% respectively, *p* < 0.001). The median duration of remission (DOR) was approximately 1.5 months longer in the inotuzumab group compared to the standard chemotherapy group (4.6 vs. 3.1 months, *p* = 0.03).

Regarding safety, the most common treatment‐emergent AEs in both groups were hematologic, with cytopenias reported frequently. Serious AEs were similar between the two arms, with febrile neutropenia being the most common (12% vs. 18% in inotuzumab and chemotherapy arms, respectively). Hepatotoxicity, including VOD, was more frequent in the inotuzumab group (11% vs. 1%), particularly among patients who proceeded to alloSCT, where 10 out of 48 developed VOD, including 2 fatalities [21].

A subsequent long‐term follow‐up of the INO‐VATE study confirmed the survival advantage of inotuzumab [22]. Two‐year OS rates were 22.8% in the inotuzumab arm and 10.0% in the chemotherapy arm (*p* = 0.015). Subgroup analysis indicated that nearly all patient subgroups derived benefits from inotuzumab, including those with unfavorable baseline characteristics such as advanced age or short duration of first remission. In this last follow‐up analysis, factors associated with improved OS included achieving CR/CRi, MRD negativity, higher baseline platelet count and haemoglobin level, longer duration of first remission, as well as consolidation with alloSCT. No substantial change was detected in terms of the safety profile [22].

#### 2.1.3 Post Hoc Analyses of INO‐VATE Study

Following the publication of INO‐VATE results, many post hoc analyses have been conducted to identify the patient population that may derive the greatest benefit from this drug. These analyses aim to elucidate specific factors or subgroups within the RR B‐cell ALL population that could experience improved outcomes with inotuzumab. One of them evaluated the impact of disease burden on treatment outcomes [36]. Patients were stratified into low (BM blasts > 50%), moderate (50%–90%), and high (> 90%) blast disease burden groups. Inotuzumab improved survival across all subgroups, with a hazard ratio of 0.64 (*p* = 0.0260), 0.81 (*p* = 0.1109), and 0.60 (*p* = 0.0335) for low, moderate, and high disease burden, respectively.

The CR/CRi rates were higher in the inotuzumab arm for all disease‐burden subgroups. Interestingly, hepatotoxicity, including VOD, appeared less frequent in patients with high disease burden treated with inotuzumab [36].

This study also included patients with lymphoblastic lymphoma (LBL) (11 in the inotuzumab and 6 in SOC arms) or extramedullary disease (EMD) (7 in inotuzumab and 5 in SOC). Among these patients, treatment with inotuzumab was associated with improved outcomes. The CR/CRi rate was 66.7% vs. 18.2%, *p* = 0.0144 in inotuzumab and SOC groups, respectively. Moreover, patients treated with inotuzumab showed a significantly better PFS (4.4 vs. 1.6 months in inotuzumab and SOC groups respectively, *p* = 0.041) [36].

The efficacy of inotuzumab in this setting is further corroborated by a retrospective study evaluating the efficacy of inotuzumab in patients treated at MD Anderson Center for B‐ALL with EMD. After 2 cycles of inotuzumab, the ORR was 84%, with a CR rate of 55%. After a median follow‐up of 29 months, the median OS was 12.8 months and was not associated with age whether considered as a continuous variable or dichotomized at 60 years. Notably, three patients experienced sinusoidal obstructive syndrome (SOS), including one patient after undergoing transplantation [37].

Another post hoc analysis stratified patients in 2 different age cohorts (< 55 years and ≥ 55 years). In both groups, the median duration of inotuzumab therapy and the types and frequencies of AEs were generally similar. Efficacy analysis showed comparable CR, median DOR, and PFS in both age cohorts. However, the median OS was longer in younger patients (median, 8.6 vs. 5.6 months; hazard ratio, 0.61). As expected, the incidence of VOD was higher in older patients (41% vs. 17%) underlying the worse toxicity profile of these patients [38].

Concerning patients with specific molecular profiles, a specific analysis of Ph‐positive RR ALL patients from the INO‐VATE and Phase 1–2—1010 studies, inotuzumab demonstrated superior MRD negativity rates (81% vs. 33%) and CR/CRi rates (73% vs. 56%) compared to chemotherapy. Event‐free survival (EFS) at 12 months was also higher in the inotuzumab group (20.1% vs. 4.8%), although OS was not significantly different (8.7 vs. 8.4 months) [20, 21, 23].

Another post hoc study of 91 patients (43 in the inotuzumab group and 48 in the SOC group) who were evaluable for genomic profiling demonstrated a consistent advantage of inotuzumab across all leukemic subtypes, genomic alterations, and risk categories [24]. Interestingly, patients classified as having a high‐risk genomic profile—defined by the presence of Ph‐like, *KMT2Ar*, low hypodiploid, and Ph‐positive—experienced significantly higher rates of composite CR (cCR) with inotuzumab compared to SOC. In particular, inotuzumab was associated with a markedly improved CR rate in Ph‐like ALL (85.7% [6/7] vs. 0% [0/5]; *p* = 0.0076) and in those harboring *TP53* alterations (100% [5/5] vs. 12.5% [1/8]; *p* = 0.0047). This improvement in response rates correlated with a significantly longer PFS in the Ph‐like ALL subgroup. However, although patients with *KMT2Ar* and hypodiploidy also showed a trend toward higher response rates, this did not translate into a statistically significant PFS benefit, suggesting that while these subgroups may respond to inotuzumab, they remain at high risk of relapse [24].

#### 2.1.4 Inotuzumab in MRD‐Positive Patients

Recently a phase 2 study explored the safety and efficacy of inotuzumab ozogamicin in MRD‐positive patients. Twenty‐six patients (19 in 1st CR and 7 in 2nd or more advanced CR) were enrolled and received inotuzumab ozogamicin 0.6 mg/m^2^ on day 1 and 0.3 mg/m^2^ on day 8 of cycle 1, then 0.3 mg/m^2^ on days 1 and 8 of cycles 2–6. After a median follow‐up of 24 months, the 2‐year relapse‐free survival (RFS) and OS were 54% and 60%, respectively. Eighteen patients (69%) achieved a negative MRD status. Most adverse events were low‐grade; no unexpected adverse events occurred. SOS was noted in 2 patients (8%) both had a Ph‐positive ALL on concurrent ponatinib [25].

Inotuzumab was subsequently approved in August 2017 by the U.S. Food and Drug Administration (FDA) and in January 2018 by the European Medicine Agency (EMA) as monotherapy for the treatment of adult patients with RR Ph‐negative CD22‐positive ALL or Ph‐positive RR ALL that failed treatment with at least one TKI.

### 2.2 Blinatumomab

Blinatumomab is a bispecific T‐cell engager antibody construct that simultaneously binds CD3 on cytotoxic T cells and CD19 on B cells (Figure 1), thereby redirecting cytotoxic T cells to target and eliminate malignant B cells [26].

#### 2.2.1 Phase 2 Studies

A phase 2 study investigated blinatumomab in 36 RR ALL [27]. Patients received blinatumomab in cycles of 4‐week continuous infusion followed by a 2‐week treatment‐free interval. The median OS was 9.8 months, and the median RFS was 7.6 months. Twenty‐five patients (69%) achieved a CR or CR with partial hematological recovery (CRh), with 88% of these responders attaining MRD negativity. Of the responders, 52% (13 patients) underwent alloSCT. The most frequent AE was pyrexia, mostly in grade 1–2. Cytokine release syndrome (CRS) occurred in two patients (5.5%) and neurologic or psychiatric AEs were reported in six patients (16.7%), all of which were reversible [27].

In another multicenter, single‐arm, open‐label phase 2 study, adult patients with Ph‐negative RR ALL [39] and advanced disease (e.g., first relapse within 12 months of remission, relapse after alloSCT, or no response to or relapse after first salvage therapy or beyond, or primary refractory disease) were treated with blinatumomab. Patients received blinatumomab in cycles of 4‐week continuous infusion (9 μg/day for the first 7 days, followed by 28 μg/day) with a 2‐week treatment‐free interval. A total of 189 patients were treated, and 43% (81 patients) achieved CR/CRh after the 2 cycles of therapy. Among responders, 40% (32 patients) underwent alloSCT. Grade ≥ 3 AEs included febrile neutropenia (25%), neutropenia (16%), and anemia (14%). Grade 3 CRS was reported in 2% of patients, and grade 3 or 4 neurologic events were reported in 11% and 2% of patients, respectively [39].

#### 2.2.2 TOWER Trial

The phase 3 study TOWER trial further explored the efficacy of blinatumomab in RR advanced ALL patients [28]. This multi‐institutional study randomly assigned 405 patients (271 to blinatumomab and 134 to standard chemotherapy) in a 2:1 ratio to receive blinatumomab or SOC. Among 376 patients who received at least one dose, the median OS resulted significantly longer in the blinatumomab group (7.7 vs. 4 months; *p* = 0.01). The 6‐month EFS rate was also significantly better with blinatumomab (31% vs. 12%; *p* < 0.001), along with a longer median DOR (7.3 vs. 4.6 months).

After 12 weeks of treatment, the CR/CRh/CRi rate was 44% in the blinatumomab group compared to 25% in the standard chemotherapy group (*p* < 0.001). The benefit of blinatumomab in the CR rate was consistent across all subgroups analyzed.

Among the patients who responded, 76% in the blinatumomab group achieved MRD negativity, compared to 48% in the chemotherapy group.

Grade ≥ 3 AEs were reported in 87% of the blinatumomab group and 92% of the chemotherapy group. In particular, grade > 3 neurologic events occurred in 9.4% of the blinatumomab group and 8.3% of the chemotherapy group. Grade ≥ 3 CRS was reported in 4.9% of the blinatumomab group and 0% of the chemotherapy group [28].

A post hoc analysis of patients enrolled in this study evaluated the efficacy of blinatumomab in RR Ph‐like ALL [40]. Of the 142 samples analyzed, 15 patients (11%) were identified as having Ph‐like ALL (nine in the blinatumomab group and six in the SOC group). At week 12, the remission rates were comparable between patients with (*n* = 9) and without (*n* = 92) Ph‐like ALL. Among patients in the blinatumomab group who achieved a cCR, two of four (50%) patients with Ph‐like and 18 of 43 (42%) patients without Ph‐like ALL achieved a complete MRD response. In contrast, none of the patients with Ph‐like ALL who achieved a cCR in the SOC group attained a complete MRD response. Median OS was similar between patients with and without Ph‐like ALL treated with blinatumomab. The safety profile was also comparable, with neurologic AEs being the most frequently observed in both treatment arms. These findings suggest that blinatumomab may help mitigate the historically dismal prognosis associated with BCR‐ABL1‐like ALL. However, given the limited sample size, further studies are necessary to validate these results [40].

#### 2.2.3 Blinatumomab as Consolidation Therapy

Considering its efficacy in the achievement of deep and durable responses, blinatumomab was further valued in a phase 2 study involving 116 adult ALL patients who achieved CR but not MRD negativity [41]. Patients received blinatumomab 15 μg/m^2^/day for up to 4 cycles, with the option of alloSCT after the 1st cycle. After a median follow‐up of 29.9 months (focusing on 110 Ph‐negative patents), the median RFS was 18 months, with better outcomes in those treated in the first CR (24.6 months vs. 11 months), as well as in those who achieved MRD negativity (23.6 months vs. 5.7 months). Similarly, the MRD negativity status predicted a longer OS (38.9 months vs. 12.5 months). Notably, no additional MRD responders were observed after subsequent cycles.

In total, 78% of the 88 patients who had evaluable MRD markers achieved a complete MRD response after the first cycle, with two additional patients achieving MRD negativity after the second cycle. Notably, no additional MRD responders were observed after subsequent cycles. Among five Ph‐positive patients, three achieved MRD response after the first cycle.

AEs were consistent with previous studies, with 53% of patients experiencing neurologic events of any grade, and 10% and 3% experiencing grade 3 and 4 neurologic events, respectively. CRS (two grade 1, two grade 3) were observed in 4 patients (3%), all during cycle 1 [41].

Another phase 3 study evaluated the role of blinatumomab monotherapy as consolidation therapy in patients with Ph‐negative ALL who achieved MRD‐negativity—defined as < 0.01% leukemic cells in bone marrow in flow cytometry—following induction and intensification chemotherapy [42]. Patients were randomized to receive 4 cycles of consolidation chemotherapy alone or in combination with 4 cycles of blinatumomab. A total of 224 patients were enrolled, with 112 in each treatment arm.

After a median follow‐up of 43 months, patients in the blinatumomab group showed significantly, improved outcomes compared to those receiving chemotherapy alone. The 3‐year OS was 85% vs. 68% (HR 0.41), and the 3‐year RFS was 80% vs. 64% (HR 0.53), respectively. The survival benefit associated with the addition of blinatumomab was observed across all age groups, including both younger (< 55 years old) and older patients (> 55 years old) [42].

Notably, the study categorized patients into three prognostic risk groups according to molecular and cytogenetic profiles: favorable risk (high‐hyperdiploid, DUX4‐rearranged, TCF3‐PBX1 or PAX5 P80R), intermediate risk (PAX5‐altered, PAX5‐ETV6, ZNF384‐rearranged or MEF2D‐rearranged), and unfavorable risk (low‐hypodiploid, near‐haploid, *KMT2A*‐rearranged, Ph‐like, high‐hyperdiploid with Ph‐like, *BCL2*‐rearranged, *MYC*‐rearranged, ETV6‐RUNX1‐like with IGH‐CRLF2 fusion or CRLF2‐rearranged). In a post hoc subgroup analysis, the addition of blinatumomab to consolidation treatment conferred a survival advantage in OS and RFS across all three risk groups [42].

#### 2.2.4 ALCANTARA Study (Philadelphia‐Positive ALL)

Since the TOWER trial excluded patients with Ph‐positive ALL, the phase 2 ALCANTARA study specifically addressed this group [43]. Forty‐five Ph‐positive ALL patients received 2 cycles of monotherapy blinatumomab, followed by up to three consolidation cycles. Sixteen patients (35.6%) achieved CR/CRh by the end of the second cycle. After a median follow‐up of 16.1 months, the median RFS was 6.8 months. After 25.1 months of follow‐up, the median OS was 9.0 months, with a significant OS benefit for those who achieved CR (19.8 months vs. 6 months). The median OS was not reached in patients who achieved MRD negativity. The median OS was not impacted by censoring for alloSCT.

Among the 16 CR/CRh patients, 14 achieved MRD negativity, and the median duration of MRD response was 9.7 months. The treatment‐related AEs were consistent with those previously reported [43].

#### 2.2.5 NEUF Study (Real‐World Data)

The NEUF study was a retrospective, observational study of 249 adult patients with B‐cell ALL treated with blinatumomab for MRD‐positive or RR disease [29]. Of the 109 patients treated in CR with positive MRD, 88% achieved an overall MRD response (complete or incomplete MRD response) after the first cycle. The MRD response rates did not differ between first or later CR patients in both the Ph‐negative (88% and 91%, respectively) and the Ph‐positive subgroups (57% and 56%, respectively). After a median follow‐up of 18.5 months, the median OS was not reached. The median disease‐free survival (DFS) was 27.6 months with better outcomes for Ph‐positive patients. Both OS and DFS were longer in the first CR cases compared to those with later CR.

In the RR disease cohort, 51% of Ph‐negative patients and 41% of Ph‐positive patients achieved cCR within 2 cycles of blinatumomab, with 83% and 67%, respectively, achieving MRD response. The median OS for Ph‐negative patients was 12.2 months (9.5 months when censoring for alloSCT), and the median RFS was 11.0 months, regardless of censoring for alloSCT. In the Ph‐positive subgroup, the median OS was 16.3 months, with consistent results regardless of censoring for alloSCT. The median RFS for Ph‐positive patients was 6.7 months, similarly unaffected by additional censoring for alloSCT. In this study, the safety profile, including the incidence of CRS and neurologic events, was consistent with those reported in clinical trials [29].

Given these positive outcomes, blinatumomab was approved by the U.S. FDA in December 2014 for RR ALL and in March 2018 for MRD‐positive ALL. EMA approved blinatumomab use in November 2015 for Ph‐negative RR ALL and in January 2019 for patients with MRD‐positive ALL.

#### 2.2.6 Subcutaneous Formulation

A new subcutaneous formulation was developed to further improve efficacy and simplify blinatumomab administration. A phase 1b study explored the safety, efficacy, and pharmacokinetics of blinatumomab in heavily pre‐treated adults with RR ALL [30]. In the dose expansion phase, patients were assigned to one of four dosing cohorts: cohort 1, 40 mcg/day (QD) in the first week, followed by 250 mcg three times a week (TIW); cohort 2, 120 mcg/day (QD) in the first week, followed by 250 mcg three times a week (TIW); cohort 3, 250 mcg/day (QD) the first week followed by 500 mcg three times a week (TIW); and cohort 4, 500 mcg/day (QD) for the first week and 1000 mcg three times a week (TIW). In the expansion phase, patients received SC blinatumomab at either 250/500 mcg or 500/1000 mcg dosing regimens. At the data cut‐off, 29 patients had been treated: 14 at the 2 were 50/500 mcg dose and 13 at the 500/1000 mcg dose.

After 2 cycles, the CR/CRh rates were 85.7% in the 250/500 mcg cohort, with 75% achieving MRD negativity, and 92.3% (12/13 patients in the 500/1000 mcg cohort) with all achieving MRD negativity.

AEs were reported in all patients, with grade ≥ 3 AEs observed in 85.7% and 61.5% of patients in the 250/500 mcg and 500/1000 mcg cohorts, respectively. Grade ≥ 3 CRS occurred in 21.4% and 23.1%, while grade ≥ 3 neurologic events were seen in 42.9% and 23.1%, respectively. The median duration of grade ≥ 3 CRS was 2 days, and for grade ≥ 3 neurologic events, it was 7 days. No grade 4 CRS or neurologic events were reported. The rates of drug interruption or discontinuation were lower in the 500/1000 mcg cohort (69.2%) compared to the 250/500 mcg group (78.6%) [30].

Resistance to blinatumomab, particularly due to CD19 loss, remains a significant challenge. CD19 loss is more frequently observed in patients who relapse after CAR‐T therapy [31, 44], but it is less commonly reported after blinatumomab treatment [32, 45].

## 3 Combination Strategies

### 3.1 Combination Therapies With Inotuzumab

The development of combination therapies involving inotuzumab ozogamicin has shown promising results in enhancing treatment efficacy for RR ALL. Studies integrating inotuzumab with low‐intensity chemotherapy regimens, such as mini‐hyper‐CVD, dose‐adjusted EPOCH (DA‐EPOCH), or the Bcl‐2 inhibitor venetoclax, have explored strategies to optimize response rates and deepen remission while balancing toxicity. These regimens aim to leverage synergistic effects between targeted and cytotoxic agents to improve outcomes in this challenging clinical setting [46, 47, 48].

#### 3.1.1 Inotuzumab With Mini‐Hyper‐CVD

A phase 2 study evaluated inotuzumab combined with low‐intensity chemotherapy (mini–hyper‐CVD) in 59 patients with RR ALL [46]. The regimen included cyclophosphamide and dexamethasone (50% dose reduction), methotrexate (75% dose reduction), and cytarabine (0.5 g/m^2^ × 4 doses). Inotuzumab was administered on day 3 of the first 4 cycles (1.8–1.3 mg/m^2^ for cycle 1; 1.3–1.0 mg/m^2^ for subsequent cycles). After a median follow‐up of 24 months, the median RFS and OS were 8 and 11 months, respectively. The 1‐year OS rate was 46% overall, with 57%, 26%, and 39% for patients treated in salvage 1, salvage 2, and salvage 3 or beyond, respectively. The ORR was 78%, with a CR rate of 59%. Among responders, 82% achieved MRD negativity.

Common grade 3–4 AEs included prolonged thrombocytopenia (81%), infections (73%), and hyperbilirubinemia (14%). VOD occurred in 15% of patients [46].

#### 3.1.2 Inotuzumab With DA‐EPOCH

A phase 1 study combined Inotuzumab ozogamicin with dose‐adjusted etoposide, prednisone, vincristine, cyclophosphamide, and doxorubicin (DA‐EPOCH) in 24 patients with a median of three prior therapies [47]. Inotuzumab was administered at three dose levels on days 8 and 15 of each 28‐day cycle, after chemotherapy, which was administered on days 1 to 5. Dose‐limiting toxicity occurred in 27% of patients at the highest dose (0.6 mg/m^2^). The CR rate was 84%, with 88% achieving MRD negativity. Median OS, DOR, and RFS were 17, 15, and 9.6 months, respectively [47].

#### 3.1.3 Inotuzumab With Venetoclax

A phase 1 study explored the safety and efficacy of inotuzumab in combination with the Bcl2 inhibitor venetoclax in nine patients with RR ALL (7 Ph‐negative and 2 Ph‐positive) [48].

Venetoclax was administered with a 3‐day ramp (DL1: 100/200/200 mg; DL2: 100/200/400 mg) followed by an induction cycle consisting of inotuzumab on days 1, 8, and 15 (0.8/0.5/0.5 mg/m^2^) and daily oral venetoclax on days 1–21. Patients without a morphologic CR after the first cycle could receive a second induction, while those achieving CR proceeded to up to four 28‐day consolidation cycles (inotuzumab 0.5 mg/m^2^ on days 1, 8, 15 and venetoclax on days 1–21). All enrolled patients achieved CR, with 87% (7/9) attaining MRD negativity.

In DL1, three patients were enrolled without experiencing DLT. All achieved MRD‐negative CR and proceeded to consolidation. In DL2, three patients were enrolled, also without DLTs. All achieved MRD‐negative CR, with two proceeding to consolidation, while one patient with *KMT2A rearrangement* experienced an early relapse with lineage switch. A confirmatory cohort of three additional patients at DL2 also reported no DLT. All achieved CR, with two attaining MRD negativity. The remaining patient transitioned to blinatumomab as a bridge to transplant after 2 cycles of venetoclax and inotuzumab.

After a median follow‐up of 195 days, two patients relapsed after 48 and 177 days, respectively. Grade 3–4 AEs included febrile neutropenia, hypotension, hypertension, tumor lysis syndrome, and disseminated intravascular coagulation. No cases of VOD/SOS were reported [48].

### 3.2 Combination Therapies With Blinatumomab

Recent studies have focused on combining blinatumomab with TKIs (especially ponatinib) or with venetoclax, a Bcl‐2 inhibitor, in CD19‐positive Ph‐negative ALL. These combinations seek to exploit complementary mechanisms of action to achieve deeper remissions, overcome resistance, and expand treatment options for high‐risk patient populations.

#### 3.2.1 Blinatumomab With TKIs

In 2017, the MD Anderson group published a retrospective analysis of 12 adults with RR Ph‐positive ALL (9 patients) and chronic myeloid leukaemia in lymphoid blast crisis (LBC‐CML) (3 patients) who received blinatumomab in combination with TKI (8 ponatinib, 3 dasatinib, and 1 bosutinib). Six patients were in hematological relapse, and 6 patients were in persistent MRD positivity [33].

After a median follow‐up of 8 months, the median OS was not reached. The complete hematologic, cytogenetic, and molecular response rates were 50% (3/6), 71% (5/7), and 75% (9/12), respectively. No patients reported cardiovascular events, while 2 cases of CRS were reported, all reversible [33].

In 2021, another retrospective study evaluated the efficacy of combining blinatumomab and ponatinib in 26 RR Ph‐positive ALL patients [49]. Twenty‐five out of 26 patients (96.2%) achieved a CR, and 23 achieved a complete molecular response. Notably, two patients with morphologic CR experienced isolated CNS progression but responded to intrathecal chemotherapy and subsequently continued ponatinib maintenance. Despite these cases, the CR rate was significantly higher than those reported in the ALCANTARA and PACE trials, in which patients received blinatumomab and ponatinib monotherapy, respectively [43, 49, 50]. After a median follow‐up of 34.4 months, the median OS and EFS were 20 and 15.3 months, respectively. Eight patients proceeded to alloSCT, while 15 of the 18 patients who did not undergo alloSCT continued maintenance with ponatinib monotherapy. The combination regimen exhibited a favorable safety profile, with neurological AEs reported in eight patients and CRS observed in three cases [49].

More recently, this combination was evaluated in a phase 2 study enrolling patients with newly diagnosed [treatment naïve (TN)] or RR Ph‐positive ALL, as well as those with chronic myeloid leukaemia in lymphoid blast phase (CML‐LBC) [51]. Patients received orally ponatinib at a dose of 30 mg in combination with continuous intravenous blinatumomab (28 μg/day for 28 days each cycle) for up to 5 cycles, followed by ponatinib monotherapy. A total of 60 patients were included in the study (40 TN, 14 RR, and 6 CML‐LBC). After a median follow‐up of 16 months, 33 of 38 evaluable TN patients (87%) achieved a CR. Among RR patients, 12 of 13 evaluable patients (92%) demonstrated an overall response, with 11 patients (79%) achieving a CMR. Similarly, 5 of 6 CML‐LBC patients (83%) exhibited an overall response.

Concerning the safety profile, the most frequently reported grade 3–4 AEs included infection, elevated pancreatic and liver enzymes, pain, and hypertension. Blinatumomab was discontinued in one patient due to persistent grade 2 tremor, while ponatinib was discontinued in three patients due to cerebrovascular ischemia, portal vein thrombosis, and coronary artery stenosis. Importantly, no treatment‐related deaths were reported [51].

#### 3.2.2 Blinatumomab With Venetoclax (BLIVEN Trial)

In the BLIVEN phase 1 trial, seven adult RR or MRD‐positive CD19‐positive Ph‐negative BCP‐ALL received oral Venetoclax on day 7 to day 42 of target doses ranging between 200 and 800 mg daily and blinatumomab as a continuous intravenous infusion of 28 mcg/day (adjusted for RR patients) 4 weeks on and 2 weeks off, starting on day 1 [52]. At the enrolment date, three patients were in hematologic relapse and four with MRD‐positive diseases. Venetoclax target doses were 400 mg (*n* = 3), 600 mg (*n* = 3), and 800 mg (*n* = 1).

Among three RR patients, one achieved CR with MRD negativity. Among four MRD‐positive patients, three attained MRD negativity [52]. No dose‐limiting toxicities were observed, and the maximum tolerated dose (MTD) was not reached. The regimen showed an acceptable safety profile with no treatment interruptions during the first cycle [52]. These combination strategies highlight the potential to optimize outcomes with targeted chemotherapy regimens in R/R ALL while maintaining manageable toxicity profiles.

## 4 Beyond Blinatumomab and Inotuzumab

Other monoclonal antibodies have been investigated in the treatment of RR ALL, although clinical responses remain limited.

A phase 1 study explored the safety and efficacy of the radioimmunoconjugate ^90^Y‐DOTA epratuzumab, administered as monotherapy on days 1 and 8 (± 2 days) across four dose levels [53]. Seventeen patients were enrolled, with only one DLT (aplasia lasting 8 weeks) occurring at the highest dose level. The maximum tolerated dose was not reached. The most common grade 3–4 AEs were pancytopenia and infections. Two patients reached CR at 5 and 6 weeks, while one patient achieved CRi at 8 weeks. No partial responses were reported, and none of the responders proceeded to alloSCT. The median OS was 109 days, and the median leukemia‐free survival (LFS) for responders was 185 days [53].

A phase 2 study investigated the anti‐CD19 antibody‐drug conjugate coltuximab ravtansine in 36 patients [54]. Three dose levels (55, 70, and 90 mg/m^2^) were tested during dose escalation (19 patients) and expansion (17 patients). One DLT (grade 3 peripheral neuropathy) was reported at the dosage of 90 mg/m^2^. The 70 mg/m^2^ dose was selected for the expansion phase. The most common AEs were pyrexia, diarrhoea, and nausea. Among 17 evaluable patients, four achieved a response. Due to the low clinical response rate, the study was prematurely discontinued [54].

Another phase 2 study evaluated the anti‐CD19 monoclonal antibody tafasitamab as monotherapy in 22 patients with RR ALL [55]. The ORR was 9%, with 16 patients (73%) progressing before their first response assessment. Responses were short‐lived, lasting 8 weeks in a patient with CR and 4 weeks in a patient with MRD‐negative CRi. The most common AEs of any grade were infusion‐related reactions (59.1%) and fatigue (40.9%), while the most frequent grade 3–4 hematologic AE was febrile neutropenia (22.7%) [55].

CD‐123 has emerged as a novel therapeutic target of interest due to its widespread expression across many haematological malignancies. A phase 1 study evaluated the safety and efficacy of flotetuzumab, a CD123‐targeting agent, in RR ALL (Cohort A) and other advanced CD123‐positive non‐myeloid hematological malignancies (cohort B). In Cohort A, 9 patients with RR ALL received flotetuzumab at a dose of 500 ng/kg/day. No dose‐limiting toxicities were reported within this cohort; however, the treatment failed to elicit any objective responses [56].

Moxetumomab pasudotox, an anti‐CD22 immunotoxin, was t investigated in a phase 2 study in pediatric patients with RR ALL. Among 32 enrolled patients, 30 received the study drug and were evaluable for safety, while 28 were assessable for response. The ORR was 28.6%, comprising 3 patients (10.7%) achieving morphologic CR and five (17.9%) attaining partial responses. Despite these responses, 11 patients (39.3%) progressed during treatment. AEs were reported in 10 patients (33.3%), including 6 cases of capillary leak syndrome (CLS), 4 cases of hemolytic uremic syndrome (HUS), and 1 treatment‐related death due to pulmonary oedema [57].

The anti‐CD20 monoclonal antibody rituximab has demonstrated efficacy in the frontline treatment of adult patients with CD20‐positive ALL [58]. In the RR ALL setting, rituximab has been incorporated into multidrug regimens, including inotuzumab, blinatumomab, and low‐intensity chemotherapy, yielding promising results in pediatric patients [59].

## 5 Sequential Use of Monoclonal Antibodies as Monotherapy or Associated With Chemotherapy

Another unresolved issue is whether a sequential use of these antibodies in subsequent relapses is viable. An Italian retrospective study evaluated the sequence of blinatumomab followed by inotuzumab (Blina/InO) versus inotuzumab followed by blinatumomab (InO/Blina) in 71 RR ALL patients [60]. In the Blina/InO sequence, 36 patients (63%) achieved CR after blinatumomab, with 42% attaining MRD negativity. Following treatment with inotuzumab, 47 patients (82%) achieved CR, including 34 patients with MRD‐negative status. The InO/Blina sequence (14 patients) resulted in a median OS of 19 months, with disease‐free survival (DFS) of 7.4 months after blinatumomab (11.6 vs. 2.7 months in MRD‐negative vs. MRD‐positive patients, *p* = 0.03) and 5.4 months after inotuzumab. The Blina/InO sequence (57 patients) yielded a median OS of 9.4 months, with a DFS of 5.1 months after inO and 1.5 months after Blina (8.7 vs. 2.5 months in MRD‐negative vs. MRD‐positive, *p* = 0.02). Interestingly, OS and DFS were superior in patients who relapsed post‐alloSCT, possibly due to the role of potentially reflecting a graft‐versus‐leukemia effect mediated by donor CD3 lymphocytes, even if the effect of previous monoclonal antibodies on CD3 donor lymphocytes needs to be further investigated. The median OS from the first immunotherapy was longer in the Blina/InO cohort than in the InO/Blina group, without a statistical significance (21.9 months vs. 15.3 months, respectively, *p* = ns) [60]. These data suggest that the sequential use of inotuzumab and blinatumomab is feasible without significant loss of efficacy, and no difference in OS was observed in the two groups (Ino/Blina and Blina/Ino). So, considering that both combinations are feasible and effective when selecting immunotherapy, it is important to consider the availability of these drugs according to local regulatory authorities, the possible AEs related to them, disease‐specific factors (such as surface expression of CD19 and CD22), disease burden, and the possible involvement of extramedullary sites [2].

To further support the effectiveness of a sequential approach, a retrospective analysis of the pediatric hospital “Bambin Gesù” explored the outcomes of pediatric, adolescent, and young adult patients who underwent alloSCT for RR ALL that received blinatumomab as the last antileukemic treatment. Seventy‐eight patients were evaluated. After a median follow‐up of 23.2 months, the 2‐year DFS and OS were 72.2% and 89.2%, respectively. Of note, among patients transplanted in second or more advanced CR, those who had received the sequential combination of inotuzumab and blinatumomab had a significantly lower cumulative incidence of relapse as compared to those who did not receive inotuzumab (9.5% vs. 40.4%, *p* = 0.023), suggesting that the sequential use of both inotuzumab and blinatumomab could exert a synergistic effect in achieving and maintaining a deep response [61].

The combination of these inotuzumab and blinatumomab with chemotherapy has also been explored. A phase 2 study investigated inotuzumab combined with mini hyper‐CVD for the first 4 courses of treatment, later amended to include four consolidation cycles of blinatumomab [62]. Responders continued maintenance therapy with a regimen of prednisone, vincristine, 6‐mercaptopurine, and methotrexate, supplemented with blinatumomab every three maintenance cycles. Among 110 enrolled patients (67 pre‐amendment, 43 post‐) the ORR was 83% and the CR rate was 63%. MRD negativity was achieved by 82% of responders. After a median follow‐up of 48 months, the median OS was 17 months, with a 3‐year OS of 4% for patients treated pre‐amendment and 52% for those treated post‐amendment.

The study also included high‐risk patients, with poor prognosis observed in those harboring *TP53* mutations (3‐year OS rate of 9%) compared to those with wild‐type status (3‐year OS rate of 63%) or adverse cytogenetic features [62].

## 6 Discussion

The treatment landscape for RR ALL has undergone significant evolution, offering a range of options including monoclonal antibodies and, increasingly, cellular therapies such as CAR‐T cells. However, the optimal selection, sequencing, and integration of these modalities are topics of debate.

Currently, the European Leukemia Net does not provide specific recommendations favoring one monoclonal antibody over another due to the absence of direct comparative studies. In practice, clinical parameters often guide treatment choice: blinatumomab is generally preferred for patients with low disease burden and preserved T‐cell function, whereas inotuzumab may be more effective for those with a high disease burden or extramedullary involvement [2.25–26.33].

Randomized head‐to‐head trials comparing inotuzumab and blinatumomab are unavailable, but indirect analyses have yielded some insights. Proskorovsky and colleagues conducted a matching‐adjusted indirect comparison (MAIC) and a simulated treatment comparison (STC) using patient‐level data from the INO‐VATE and TOWER studies. Their analysis showed higher remission rates and increased likelihood of subsequent transplant with inotuzumab compared to blinatumomab, irrespective of the type of indirect treatment comparison (ITC) method used. Moreover, inotuzumab showed a trend toward improved EFS, though no differences in OS were observed [63].

Conversely, Song and colleagues performed a similar MAIC using data from the same studies. They found a survival benefit for blinatumomab over inotuzumab. After adjusting for baseline characteristics, the median OS was 9.3 months for blinatumomab versus 7.7 months for inotuzumab (*p* = 0.4), with a relatively restricted mean survival time (RSMT) at 12 months favoring blinatumomab by 1.6 months. However, no significant differences were observed in remission rates between the two therapies [64]. These conflicting findings underscore the complexity of interpreting results from indirect comparisons, highlighting the need for randomized studies to definitively determine the optimal antibody‐based therapy for RR ALL. Moreover, disease and patient features need to be carefully evaluated. Blinatumomab has shown sustained remissions but is less effective in patients with high disease burden, while inotuzumab has shown short remission but was effective in high disease burden and extramedullary involvement [21, 22, 28].

In recent years, the treatment paradigm for ALL has evolved, with a growing interest in increasing interest in incorporating blinatumomab, and to a lesser extent inotuzumab, into first‐line treatment regimens. This shift has raised critical questions regarding the feasibility of retreatment with the same monoclonal antibody or the use of another agent targeting the same antigen in subsequent relapses [65, 66]. Preliminary results from the GIMEMA LAL 2317 study showed a haematological CR in 131 of 146 enrolled patients (90.4%). Notably, after early consolidation, 73% of these patients achieved MRD negativity, which further increased to 96% following the first cycle of blinatumomab. Of particular interest, among 25 Ph‐like patients, 10 who were MRD positive after early consolidation converted to MRD‐negative status after blinatumomab therapy and became. These findings are consistent with the post hoc analysis of the TOWER trial, which highlighted the efficacy of blinatumomab in this high‐risk patient subset [66].

Considering their different properties, a sequential approach appears as an attractive option, but its feasibility and efficacy remain another open question. Retrospective analyses suggest that combining or sequencing inotuzumab and blinatumomab may optimize outcomes when tailored to disease burden. For instance, induction with inotuzumab, effective in high‐disease‐burden settings, followed by blinatumomab consolidation, which performs better in low‐disease‐burden contexts, represents a reasonable strategy [36, 37, 39]. Data from the INO‐VATE study highlight the importance of post‐inotuzumab consolidation to address the short duration of responses observed in patients with high disease burden. Additionally, for patients undergoing alloSCT, extending the interval between inotuzumab and transplant is crucial to reduce the risk of VOD [21, 22].

The underlying biology of ALL also plays a pivotal role in treatment selection. Both inotuzumab and blinatumomab are effective in both Ph‐positive and Ph‐negative ALL. Available data also suggest their efficacy in Ph‐like ALL, suggesting that adding a monoclonal antibody in the consolidation regimens of these patients can improve responses and reduce the risk of relapse [24, 40, 66]. However, genetic lesions, such as *TP53* mutations, remain associated with poor responses and reduced survival, even with sequential monoclonal antibody therapy [62]. These findings emphasize the urgency of developing novel therapeutic strategies to improve outcomes for high‐risk patients with adverse genetic profiles [67, 68].

The treatment of RR ALL in elderly patients unfit for alloSCT remains a significant challenge. Outcomes in this population are poor due to a higher risk of treatment‐related toxicity and the frequent presence of adverse molecular or cytogenetic features, such as *KMT2A*r, hypodiploidy, and *TP53* mutations. In these settings, monoclonal antibodies, either as monotherapy or in combination with very low‐intensive chemotherapy, sound like a very attractive option [69]. In recent years, CAR‐T therapy has raised growing interest due to its potential efficacy and relatively manageable safety profile [70].

More recently, to further enhance antitumor efficacy and activity and mitigate immune escape mechanisms, a trispecific CD19/CD22/CD3 antibody (tsAb) has been developed. This molecule facilitates immune synapse formation between neoplastic cells and T cells by targeting both CD19 and CD22 while engaging CD3 [71]. Preclinical studies in vitro and in murine models have demonstrated promising efficacy, warranting further investigation in clinical trials [71].

As monoclonal antibodies are increasingly integrated into frontline treatment protocols, determining the optimal management of relapsed patients becomes even more critical. A deeper molecular understanding of ALL will be instrumental in refining treatment strategies and enabling personalized approaches to therapy.

Continued research into novel agents, combinations, and biomarkers of resistance will further advance the goal of achieving durable remissions and improving survival in RR ALL.

## Author Contributions

All authors contributed to the manuscript and were involved in revisions and proofreading. All authors approved the submitted version.

## Conflicts of Interest

The authors declare no conflicts of interest.

## Data Availability

The authors have nothing to report.
